# Examining lifestyle factors as potential moderators of the link between childhood adversity and comorbid psychological distress and obesity in early adulthood

**DOI:** 10.1186/s12889-025-23505-6

**Published:** 2025-07-07

**Authors:** Tom Woofenden, Graeme Fairchild, Thomas Matthew Lancaster

**Affiliations:** Department of Psychology, University of Bath, Bath, Somerset, BA2 7AY UK

**Keywords:** Lifestyle factors, Childhood adversity, Depressive symptoms, Obesity, Comorbidity

## Abstract

**Background:**

Whilst childhood adversities have been shown to be risk factors for mental, physical, and comorbid health problems in childhood and middle-to-late adulthood, there is less evidence for these associations in early adulthood. It is also unclear if lifestyle factors can modify the risk of these health outcomes following childhood adversities. This study aims to examine childhood adversities as risk factors for psychological distress, obesity, and their comorbidity, and further quantify the moderating impact of lifestyle factors. This could provide insight into potential protective influences against the detrimental health consequences of childhood adversities, particularly mental-physical comorbidity.

**Methods:**

Analyses were conducted on data from the 1970 British Cohort Study (*n* = 16,407). The cumulative impact of parent- and self-reported adversities (range: 0–33) were consolidated across childhood (0–16 years) and examined as a predictor of psychological distress (Malaise Inventory ≥ 8), obesity (BMI ≥ 30 kg/m^2^) and their comorbidity in early adulthood (30 years) using multinomial logistic regression. Self-reported lifestyle factors in adolescence (16 years), including physical activity, diet, sleep duration, smoking, and alcohol consumption were assessed as moderators of the association between childhood adversities and the specified outcome categories.

**Results:**

A one-item increase on the childhood adversities scale elevated the risk of psychological distress (OR [95% CI]; 1.11 [1.09, 1.13]), obesity (OR [95% CI]; 1.05 [1.03, 1.06]) and mental-physical comorbidity (OR [95% CI]; 1.16 [1.12, 1.20]). Compared to comorbidity, childhood adversity was a weaker risk factor for psychological distress (OR [95% CI]; 0.96 [0.92, 0.99]) or obesity alone (OR [95% CI]; 0.90 [0.87, 0.94]). No lifestyle factors were significant moderators of the association between childhood adversities and these health outcomes.

**Conclusions:**

Consistent with evidence from middle- to late-adulthood, childhood adversities showed a stronger association with comorbidity in early adulthood than mental or physical health problems alone. There was no evidence that lifestyle factors influenced the association between childhood adversities and comorbidity or individual health problems. Our findings highlight the importance of considering comorbidities when investigating the negative health consequences of childhood adversities, and therefore, the continuing need to identify factors which mitigate the increased risk of comorbidity.

**Supplementary Information:**

The online version contains supplementary material available at 10.1186/s12889-025-23505-6.

## Background

The co-occurrence of mental health conditions and obesity represents a growing public health concern, due to how their co-occurrence can exacerbate the mental and physical impairments associated with each health problem in isolation [1]. Bidirectional associations between mental and physical health problems suggest shared aetiology [2, 3], and research is underway to determine common risk factors for their comorbidity [4, 5]. Childhood adversities have been shown to increase the risk of co-occurring high internalising symptoms and fat mass percentage in childhood [6] and co-occurring depression and cardiometabolic disease in adulthood [7]. These studies have documented an association between childhood adversities and an increased risk of comorbidity beyond mental and physical health problems alone in middle-to-late adulthood (40–70 years), but not in childhood (13–14 years). As an intermediate timepoint in the lifespan, it is important to examine these relationships in early adulthood (30 years). This will further our understanding of when the effect of childhood adversities on comorbidity becomes elevated beyond the impact on mental or physical health problems alone. Understanding the relationship between childhood adversities and comorbid mental and physical health problems will provide a platform to understand whether lifestyle behaviours moderate this relationship, which could highlight protective effects of these behaviours.

Although there has been some focus on childhood adversities as risk factors for mental-physical comorbidities in early adulthood, comorbidities have been defined by counts of various conditions in the mental and physical health domains [8]. However, this approach fails to describe the non-random clustering of certain conditions. Identifying non-random comorbidity clusters is important because their higher prevalence puts an added strain on healthcare systems, and identification can also help researchers understand the risk factors which lead to specific comorbidities [9]. Related to the current study, UK primary care data (from ~ 39,000,000 adults) indicates that physical health conditions are more common in individuals with mental health problems [10]. This association was higher than would be expected by chance, and particularly strong in younger adults. Within mental-physical comorbidities, depression and obesity have shown a bidirectional association, with the presence of each condition increasing the risk of developing the other [2]. As mentioned, comorbidities often cluster non-randomly due to common risk factors [11]. Within the comorbidity of depression and obesity, repeated activation of the stress response has been highlighted as a potential risk factor [12]. The identification of early common risk factors is particularly important for the comorbidity of depression and obesity, due to how each of these health problems is said to fuel a “cyclical” mechanism which is particularly difficult to break once established [12]. In examining childhood adversity as a risk factor for comorbid mental and physical health problems in early adulthood, this study aligns with the UK Chief Medical Officer’s aim of identifying common risk factors for specific disease clusters in multimorbidity [11, 13].

Childhood risk factors for the comorbidity of psychological distress and obesity have previously been investigated using data from the 1970 British Cohort Study (BCS70). Specifically, low socioeconomic status in childhood has been shown to elevate the risk of mental-physical comorbidity in early adulthood [14, 15]. However, other adversities such as abuse and neglect are known to increase the risk of mental-physical comorbidity in adulthood, even when controlling for socioeconomic status [7]. Maltreatment and other childhood adversities are strongly linked with low socioeconomic status at a population level [16]. Therefore, examining the effects of low socioeconomic status alone may not sufficiently account for the health impacts of other adversities. This was highlighted in one study which showed that low adulthood socioeconomic status partially mediated the relationships between childhood adversities and depression (8–12%) and obesity (0–15%) [17]. Other studies showed similar results, with low adulthood socioeconomic status (10%) and education (7%) mediating minor proportions of the relationship between childhood adversities and depressive symptomology [18, 19]. These findings, and the large body of evidence outlining the detrimental impact of childhood maltreatment on biological systems (e.g. hypothalamic-pituitary-adrenal (HPA) axis, inflammation, etc.) [20], highlight that the mechanisms linking childhood adversities to depression and obesity are not fully explained by socioeconomic status. Therefore, combining childhood adversities and low socioeconomic status to construct the antecedent exposure, allows for an understanding of how cumulative childhood stressors impact mental, physical, and comorbid health problems. This approach aims to establish the context in which moderator variables, i.e., lifestyle factors could mitigate health problems in a target population.

Given that childhood adversities are established risk factors for mental and physical health issues, this study prioritises identifying modifiable factors that may attenuate these elevated risks. Lifestyle behaviours, which can be influenced by early-life experiences, are also strongly linked to both later mental and physical health [21–23]. These behaviours therefore represent modifiable approaches which may attenuate pathways to mental, physical, and comorbid health problems, which warrants further exploration. We therefore sought to investigate lifestyle factors as moderator variables, which were chosen both due to their ability to be modified, and their established links with mental and physical health. Specifically, lower physical activity, poor dietary habits, sleep problems (both short and long sleep duration), cigarette smoking, and excessive alcohol consumption are all associated with an increased risk of depression [24–26]. These factors are also associated with an increased risk of obesity [27–30], although the relationship between cigarette smoking and obesity remains unclear [31, 32]. Despite evidence for a protective role of a healthy lifestyle on the risk for depression and obesity considered separately, the effects of a healthy lifestyle on their comorbidity are less conclusive [33–35]. We therefore aim to clarify whether adopting a healthy lifestyle can attenuate the link between childhood adversities and mental-physical comorbidity. We also examine whether healthy lifestyle factors alter the relationship between childhood adversities and psychological distress, or obesity considered separately.

In addition, the majority of studies investigating the influence of lifestyle factors on comorbid depression and obesity analysed cross-sectional data, making the directionality of relationships difficult to determine [35]. Here, we developed a cumulative measure of childhood adversities (0–16 years) as an antecedent exposure, studied lifestyle factors as moderator variables (16 years), and examined their conditional effects on psychological distress, obesity, and their comorbidity as outcomes (30 years). The temporal ordering of variables allowed us to infer directionality in the pathway from childhood adversities and lifestyle factors to health outcomes. Specifically, lifestyle factors at 16 years were chosen to determine the antecedent impact of healthy behaviours as preventative measures, rather than correlates at the same timepoint. This helps to reduce the potential confounding effect of these health problems on participation in healthy lifestyle behaviours, as mental health problems and obesity can precede changes in lifestyle behaviours [36–38]. Therefore, longitudinal measures were assessed to help establish directional pathways between childhood adversities and mental, physical, and comorbid health problems. This was followed by the examination of lifestyle behaviours as preventative measures, which may attenuate the longitudinal effects of childhood adversities and these health outcomes.

To achieve these objectives, the current study employs data from the BCS70 to investigate the association between childhood adversities and the comorbidity of psychological distress and obesity in early adulthood. This builds on established models to consider childhood adversities beyond low socioeconomic status [14, 15]. We also examine whether the effects of childhood adversities on comorbidity are greater than psychological distress or obesity alone. We further aim to assess putative moderating effects of modifiable lifestyle factors, such as physical activity, adherence to a Mediterranean diet, calorie intake, sleep duration, alcohol consumption and cigarette smoking, on the relationship between childhood adversities and mental-physical comorbidity. We capitalise on longitudinal data to examine potential factors which may confer vulnerability or protection against early adulthood mental-physical comorbidity in individuals exposed to childhood adversities. Identifying protective lifestyle factors that weaken this relationship could help mitigate the detrimental consequences of childhood adversities on mental, physical, and comorbid health problems.

## Methods

### Study participants

The 1970 British Cohort Study (BCS70) is a multidisciplinary longitudinal study of over 17,000 participants born in England, Scotland, and Wales during a single week in 1970 [39]. Data was collected at numerous waves by both parent and participant interviews, medical records, examinations, and questionnaires. Of the available sample (*N* = 16,771), twins (*n* = 340) were removed due to the potential for genetic confounding. Individuals with a physical handicap (*n* = 24) were also removed due to barriers to physical activity, deriving the final analysis sample (*n* = 16,407).

### Childhood adversities

Psychosocial stressors were summed to capture the cumulative exposure to adversities across childhood (assessed at 5, 10 and 16 years). The 33 items covered a range of life stress domains related to the family environment, socioeconomic status, abuse, and emotional neglect. Descriptions of all items are specified in Supplementary Table 1, including item dichotomisation criteria, the informant, timepoint of reporting, the percentage of exposure, and the missingness in each item. Briefly, measurements were reported by both the parents (25 items) and the children themselves (8 items) at 3 timepoints in development: ages 5 (10 items), 10 (10 items) and 16 years (13 items). Each item was dichotomised and summed to create a scale that ranged from 0 to 33, with higher scores indicating greater exposure to adversity. The scale had acceptable internal consistency in the current sample (α = 0.70).

### Lifestyle factors

Six lifestyle factors measured at 16 years of age were included in the current study, including physical activity, adherence to a Mediterranean diet, calorie intake, sleep duration, alcohol consumption and cigarette smoking.

#### Physical activity

was assessed by self-reported monthly participation in team-based (football, netball, etc.) and individual sports (swimming, gymnastics, etc.) at 16 years of age. A list of all included sports is shown in Supplementary Table 2.

#### A Mediterranean diet score

A Mediterranean diet score was derived using data from a self-reported food diary, where participants recorded the food items and portion sizes consumed over a period of 4 days [40]. According to standard food portion sizes, food items were quantified in grams per day. Food group specific cut-offs were then applied to the sum of items belonging to 5 beneficial food groups (fruit, vegetables, legumes, cereals, and fish) and 2 detrimental food groups (meat and dairy), where group cut-offs and specific items are shown in Supplementary Table 3. Low, moderate, and high consumption of beneficial food groups scored 0, 1 or 2 points respectively, while detrimental food groups were scored in reverse [41], amounting to an overall diet score that ranged from 0 to 14.

#### Calorie intake

was determined by the average of the calories consumed over the 4-day period (kcal/day).

#### Sleep duration

was calculated from the self-reported number of hours between bed and wake time on the night prior to arrival at an assessment centre.

#### Consumption of alcohol

over the previous year was self-reported as “Never drink”, “Special occasions only”, “Once a month” or “2 or more times a week”.

#### Cigarette smoking

was self-reported according to the number of cigarettes smoked per week using following categories: “Non-smoker”, “0–10”, “11–40” or “41 or more”.

### Confounding variables

Given potential influences of biological sex and prenatal stressors on child mental and physical health [42–44], sex, maternal smoking and alcohol consumption during pregnancy were included as covariates in all analysis models.

### Mental-physical comorbidity

Psychological distress was measured using the 24-item Malaise Inventory, which assesses psychological and somatic symptoms of depression and anxiety [45]. The Malaise Inventory has previously shown reliability and validity in a sample with clinical diagnoses of psychiatric disorders [46]. It exhibits good discriminatory power for clinically assessed depression (AUC = 0.87, SE = 0.035) [46], and showed good internal consistency in the present sample (α = 0.81). The 9-item version of the Malaise Inventory displayed strong correlations with the 9-item Patient Health Questionnaire (*r* = 0.71) and the 7-item Generalized Anxiety Disorder Scale (*r* = 0.74), highlighting its comparability with other common measures of depressive and anxiety symptoms [47]. Responses were self-reported on a yes/no basis by written questionnaire at 30 years of age, and the items were summed to yield a scale ranging from 0 to 24. A cut-off of 8 or more was used to classify psychological distress [48], and the individual items (e.g. do you often feel depressed?) are shown in Supplementary Table 4.

Body mass index (BMI) was derived from self-reported height and weight at 30 years as follows: $\:BMI=\frac{weight\:\left(kg\right)}{height\:({m)}^{2}}$. Based on established guidelines, a BMI of 30 kg/m^2^ or above indicated the presence of obesity [49]. Applying these cut-offs in combination gave rise to four groups or ‘outcome categories’:

1. Controls– absence of psychological distress and obesity (reference group).
2. Psychological distress only– presence of psychological distress, in the absence of obesity.
3. Obesity only - presence of obesity, in the absence of psychological distress.
4. Comorbidity– presence of both psychological distress and obesity.

### Handling missing data

All data were processed in R version 4.2.1 [50]. Missing data patterns and attrition rates in BCS70 data has been extensively documented elsewhere [51]. In the current sample, there were particularly high missingness rates in measurements of lifestyle factors at 16 years, including physical activity (71.0%), Mediterranean diet (74.4%), calorie intake (66.5%), sleep duration (71.8%), alcohol consumption (66.3%) and cigarette smoking (66.5%). In addition, only 5.8% of the sample had complete data on all 33 childhood adversity items (*n* = 952), where the distribution of missing childhood adversity items is shown in Supplementary Tables 1 and Supplementary Fig. 1. Data was assumed to be missing at random (MAR) [52], and multiple imputation was applied to address missing data [53]. To satisfy congeniality assumptions between the imputation and analyses models, all analysis variables were included in the imputation model [54]. Given the assumptions that data was MAR, meaning systematic differences between missing and observed data can be explained by observed data, auxiliary variables (i.e., variables outside of the substantive model) were included in the imputation model to help account for these assumptions [55]. In line with best practice in the imputation literature, auxiliary variables were selected based on being related to missingness, being correlated with the target variable and having low missingness rates [56, 57]. Multi-item scales were imputed at item level before passive imputation to derive scale values and subsequent categorisation if necessary [58]. Specific details of the imputation model, including missingness rates and auxiliary variables are specified in Supplementary Fig. 2 and Supplementary Table 5. Predictive mean matching from the mice package was applied to synthesise 30 imputed datasets of 60 iterations, and parameter estimates were then pooled using Rubin’s rules [57, 59]. Sensitivity analyses were also conducted on a subsample of participants with complete moderator, covariate, and outcome data. Due to various degrees of missingness across childhood adversities, prorated sum scores were calculated in participants with 80% or more complete adversity items and these were applied in the sensitivity analyses [60].

### Statistical analysis

Prior to moderation analyses, multinomial logistic regression from the nnet package [61] was used to regress mental-physical comorbidity categories onto childhood adversities and potential confounding variables, including participant sex, maternal alcohol consumption during pregnancy and maternal smoking during pregnancy. These analyses will quantify the strength of the association between childhood adversities and mental, physical, and comorbid health problems. We then conducted a series of analyses which included lifestyle factors in interaction with childhood adversities (childhood adversities*lifestyle factor) across separate multinomial regression models. We aimed to identify behaviours which mitigate the effects of childhood adversities on mental, physical, and comorbid health problems. Prior to analysis, calorie intake (kcal/day) was standardised using a z-transformation to yield interpretable regression coefficients. Following these analyses, several post hoc analyses ensued. Firstly, significant prediction of comorbidity by childhood adversities or interaction terms were followed up in multinomial models with comorbidity as the reference group, to directly compare whether their effects on comorbidity were elevated above that of psychological distress and obesity separately. Furthermore, evidence suggests that social components may be a key part of physical activity that reduces depressive symptoms in young adults [62], suggesting that engaging in team sports may have a larger beneficial effect than individual sports. We therefore capitalised on the availability of physical activity sub-categories in BCS70 data (i.e., team-based sports, individual sports, skill-based sports), to determine whether different types of physical activity have differential effects on outcomes. To account for multiple comparisons in multinomial models, false discovery rate (FDR) adjusted *p*-values (q-values) were used to determine significance (q < 0.05).

## Results

Sample characteristics were stratified by outcome category and are displayed in Table 1. At 16 years most participants were non-smokers (*n* = 11,956; 72.9%), and it was most common to consume alcohol once a week (*n* = 4,697; 28.6%). There were marginally more males in the control (*n* = 6,642; 52.2%), and obesity-only categories (*n* = 908; 55.6%), whilst there were marginally more females in the psychological distress only (*n* = 991; 55.1%) and comorbid psychological distress and obesity (*n* = 150; 56.8%). A permutation test with 10,000 iterations confirmed that the number of young adults with comorbid psychological distress and obesity (*n* = 264, 1.6%) was larger than would be expected by random sampling (*n* = 182, 1.1%; z = 15, *p* < 0.001). Regarding overall physical activity, participation in exercise at school (mean = 3.80, SD = 2.32) generally contributed more to the measure of overall physical activity than participation outside of school (mean = 2.90, SD = 1.95). Characteristics of the subsample with complete moderator, covariate and outcome data are presented in Supplementary Table 6.

**Table 1:** Characteristics of the study sample stratified by outcome category

Timepoint	Measurement	Units	Controls	PD only	Obesity only	Comorbidity
			*n* = 12,713	*n* = 1797	*n* = 1633	*n* = 264
			77.4%	11.0%	10.0%	1.6%
5–16 years	**Childhood Adversities**	Number of stressors	5.97 (3.83)	7.79 (4.25)	6.78 (3.95)	8.84 (4.34)
16 years	**Physical Activity**	Sessions per week	6.75 (3.48)	6.34 (3.24)	6.81 (3.38)	6.05 (3.37)
	**Sleep Duration**	Hours	8.41 (1.28)	8.39 (1.35)	8.41 (1.31)	8.41 (1.37)
	**Mediterranean diet**	Score	3.99 (1.85)	3.95 (1.86)	3.89 (1.83)	3.82 (1.77)
	**Calorie intake**	Kcal/day	2,637 (937)	2,597 (936)	2,529 (910)	2,563 (921)
	**Alcohol Consumption**	Non-drinker	1,006 (7.9%)	152 (8.4%)	124 (7.6%)	25 (9.5%)
		Special occasions only	3,243 (25.5%)	482 (26.8%)	431 (26.4%)	70 (26.5%)
		Once a month	1,821 (14.3%)	247 (13.8%)	230 (14.1%)	33 (12.5%)
		Once a week	3,671 (28.9%)	497 (27.7%)	455 (27.8%)	74 (28.0%)
		2 or more times a week	2,971 (23.4%)	419 (23.3%)	393 (24.1%)	62 (23.5%)
	**Cigarette Smoking**	Non-smoker	9,459 (74.4%)	1,108 (61.7%)	1,208 (74.0%)	181 (68.6%)
		0–10 per week	1,089 (8.5%)	166 (9.2%)	147 (9.0%)	22 (8.3%)
		11–40 per week	1,166 (9.2%)	258 (14.4%)	148 (9.0%)	32 (12.1%)
		41 + per week	999 (7.9%)	265 (14.7%)	129 (8.0%)	28 (11.0%)
Birth	**Sex**	Male	6,642 (52.2%)	806 (44.9%)	908 (55.6%)	114 (43.2%)
		Female	6,071 (47.8%)	991 (55.1%)	725 (44.4%)	150 (56.8%)
30 years	**Psychological Distress**	Malaise Inventory	2.53 (2.09)	10.73 (2.99)	2.65 (2.08)	10.56 (2.90)
	**Body Mass Index**	Kg/M^2^	23.91 (2.96)	23.71 (3.24)	33.32 (3.35)	33.83 (3.61)

Key: Continuous (Mean; SD) and categorical (Counts; %) data types were pooled across 30 imputed datasets. Timepoint refers to age of measurement, where childhood adversities were measured at 3 distinct timepoints (5, 10 and 16 years) and had a potential range of 0–33. PD = Psychological Distress

**Fig. 1:**
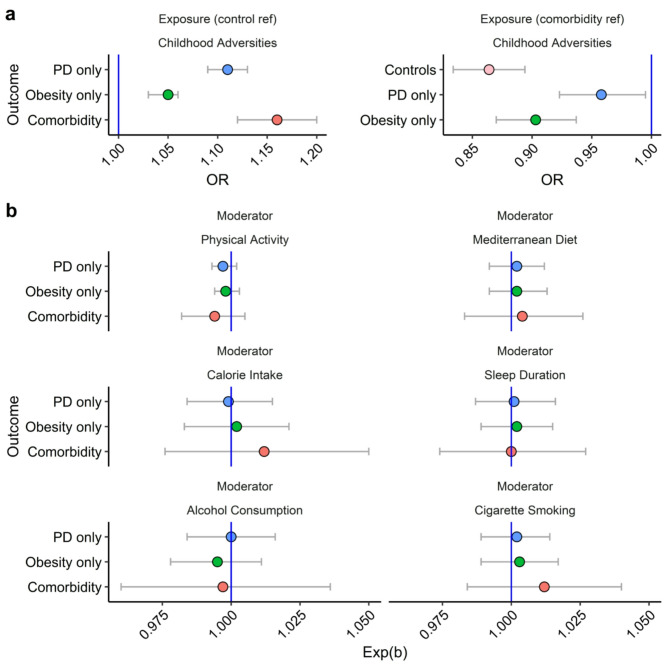
The relationship between childhood adversities and psychological distress, obesity and their comorbidity, and the influence of lifestyle factors. Forest plots display exponentiated regression betas (Exp(b)) and 95% confidence intervals from multinomial models. **a** highlights ORs for childhood adversity in multinomial models without interaction terms, with the control group as the reference category (left), and comorbidity as the reference category (right). **b** highlights exponentiated betas for interaction terms that investigate each of the lifestyle factors - physical activity, Mediterranean diet, calorie intake, sleep duration, alcohol consumption, cigarette smoking - and childhood adversities (childhood adversities*lifestyle factor) in 6 separate multinomial models. Ref = reference (i.e., reference category in multinomial logistic regression), PD = psychological distress

Prior to introducing lifestyle factors as potential moderators, the analysis showed that childhood adversities increased the odds of psychological distress (OR [95% CI]; 1.11 [1.09, 1.13], *p* < 0.001), obesity (OR [95% CI]; 1.05 [1.03, 1.06], *p* < 0.001) and comorbid psychological distress and obesity (OR [95% CI]; 1.16 [1.12, 1.20], *p* < 0.001) in multinomial logistic regression without interactions (see Fig. 1a and Supplementary Table 7). When compared to those with comorbid psychological distress and obesity, who were categorised as the reference group, childhood adversities predicted psychological distress (OR [95% CI]; 0.96 [0.92, 0.99], *p* = 0.027) and obesity only (OR [95% CI]; 0.90 [0.87, 0.94], *p* < 0.001) to a significantly lesser extent (see Fig. 1a). This demonstrated a stronger association between childhood adversities and mental-physical comorbidity than each health problem alone. Results from models on participants with complete moderator, covariate and outcome data confirmed these associations (see Supplementary Table 8).

When interpreting interaction terms in the context of our experimental design, an exponentiated beta coefficient (exp(b)) below one reflects that a lifestyle factor weakened the conditional effect of childhood adversities on an outcome category, whilst an exp(b) above 1 reflects that a lifestyle factor strengthened the conditional effect of childhood adversities on an outcome category. A q-value (a corrected *p*-value adjusted the FDR) below 0.05 signified that a moderation effect was statistically significant.

Considering this, introducing lifestyle factors as moderators did not significantly alter the strength of the association between childhood adversities and comorbid psychological distress and obesity in any model (see Fig. 1b). Specifically, there were no significant moderation effects of physical activity (exp(b) [95% CI]; 0.99 [0.98, 1.01], q = 0.460), adherence to a Mediterranean diet (exp(b) [95% CI]; 1.00 [0.98, 1.02], q = 0.854), calorie intake (exp(b) [95% CI]; 1.01 [0.98, 1.05], q = 0.706), sleep duration (exp(b) [95% CI]; 1.00 [0.97, 1.03], q = 0.995), alcohol consumption (exp(b) [95% CI]; 1.01 [0.98, 1.04], q = 0.616) or cigarette smoking (exp(b) [95% CI]; 1.00 [0.96, 1.04], q = 0.949). These findings are summarised in Fig. 1b and Supplementary Table 9, whilst results of the sensitivity analyses (confirming the lack of moderation) are presented in Supplementary Table 10.

When comparing types of physical activity, proportional to the number of activity types measured in each category, average participation in individual sports (out of 25 activities; mean = 4.46, SD = 2.62) was the most frequent type of activity session attended, followed by team sports (9 activities; mean = 2.24, SD = 1.56), and then skill-based sports (6 activities; mean = 1.15, SD = 1.21), which was only considered in post hoc analyses. Analysing these separately revealed no specific moderation effects on comorbid psychological distress and obesity for any one physical activity type, including individual sports (exp(b) [95% CI]; 0.99 [0.99, 1.00], q = 0.299), team sports (exp(b) [95% CI]; 1.00 [0.97, 1.03], q = 0.914), and skill-based sports (exp(b) [95% CI]; 1.01 [0.98, 1.04], q = 0.829). The lack of a moderation effect of lifestyle factors extended to associations between childhood adversities and the psychological distress only, and obesity only categories. The results of the sensitivity analyses confirmed these findings.

## Discussion

We first set out to establish whether childhood adversities increase the risk of psychological distress, obesity, and their comorbidity in early adulthood. We then examined several lifestyle factors as moderator variables, to determine whether adopting a healthy lifestyle can provide some protection against the mental and physical health consequences of childhood adversities.

### Childhood adversities increased the risk of comorbidity to a greater extent than psychological distress or obesity alone

We demonstrated a robust association between childhood adversities and the comorbidity of psychological distress and obesity, i.e., mental-physical comorbidity. Each reported exposure to an adversity, corresponding to a one-item increase in a cumulative measure of childhood adversities (0–33), increased the odds of developing comorbidity by 16%. After assigning comorbidity as the reference category, childhood adversities were further shown to be stronger risk factors for comorbidity than psychological distress or obesity alone. These findings align with a mega-analysis of data from middle- and older-aged adults (*n* = 156,511, 40–69 years), which found that childhood maltreatment increased the risk for the comorbidity of depression and cardiometabolic diseases to a greater extent than either condition alone [7].

Whilst the same study showed childhood maltreatment to increase the risk of comorbidity in one cohort of young adults (ALSPAC mothers; *n* = 3,927, mean age = 29 years), as far as we are aware, the current study is first in this age group to show that childhood adversities are more strongly related to the comorbidity of psychological distress and obesity than each health problem alone. These observations have implications for policy and practice. Specifically, considering mental and physical health problems beyond their individual links with childhood adversities, requiring stakeholders to consider wider effects on health. This will promote further understanding the aetiology of mental-physical comorbidity associated with childhood adversities and investigate whether comorbidity develops due to this common risk factor, or as a secondary consequence of an individual morbidity. Given the known associations between depression, obesity and cardiometabolic diseases [3, 63, 64], these results could indicate that psychological distress-obesity comorbidity in early adulthood lies on the risk pathway from childhood adversities to depression-cardiometabolic disease comorbidity. This hypothesis could be tested by mapping multimorbidity trajectories of depression, obesity and cardiometabolic disease [65], and exploring childhood adversities as predictors of these trajectory classes, where advice on predicting class trajectories is readily available [66].

The findings build upon previous analyses in BCS70 and other UK birth cohorts, demonstrating that socioeconomic disadvantage in childhood increases the risk of psychological distress-obesity comorbidity in early adulthood [14, 15]. A strong relationship between childhood maltreatment (i.e. neglect, abuse, etc.) and childhood socioeconomic status has been documented elsewhere [16], and accounting for these risks within a single scale may help capture combined effects in relation to health outcomes [67]. In examining this, this study developed a cumulative measure of childhood adversities as the main exposure, which can be utilised in future studies using the BCS70 sample. In doing so, we also show that this broader definition of adversity in childhood is a strong predictor of mental-physical comorbidity, indicating that a wider range of childhood adversities, beyond low socioeconomic status, contribute to comorbidity risk.

Despite childhood adversities being a stronger predictor of comorbidity, a 1-item increase on the adversity scale elevated the risk of psychological distress by 11% and obesity by 5% in the absence of their comorbidity. This finding conforms with other studies which found that childhood adversities increase the risk of mental and physical health problems in isolation, and in regard to these associations, the increased risk due to childhood adversities was larger for mental vs. physical health problems [6, 7]. While we used a categorical outcome variable, future research could explore continuous measures to assess whether childhood adversity shows a dose-response relationship across the distribution of psychological distress and BMI. In addition, given that the established associations between childhood maltreatment and obesity became non-significant when adjusting for current depression in a separate study [68], there is support for a hypothesis suggesting that depression makes a greater contribution than obesity to the mediating pathways between childhood adversities and comorbidity of depression and obesity [68–70]. Future studies could apply longitudinal outcome data to test this hypothesis, by applying depression as a mediator in the pathway from childhood adversities to obesity before the examining the reverse association with obesity as the mediator. Comparing these inferences would aid in our understanding of the more common temporal pathway between depression and obesity.

### Lifestyle factors did not moderate the association between childhood adversities and comorbidity or individual health outcomes

In addressing the study’s main aim, a series of moderation analyses found that contrary to our hypotheses, adhering to a healthy lifestyle did not weaken the association between childhood adversities and comorbidity. Dysfunctions in biological systems have already been highlighted as mechanisms which link childhood adversities and health problems across the life course, including under- and over-activation of stress and immune systems (inflammation, hypothalamic pituitary adrenal axis, etc.), metabolic alterations and disruptions to the microbiome [71–73]. Biological pathways to comorbidity could also be partially explained by overlapping genetic risk variants for depression and obesity [74]. As lifestyle factors did not moderate the effects of childhood adversities on our outcomes, understanding how lifestyle behaviours influence intermediate mechanisms which link our exposure and outcome would be beneficial. In addition, children exposed to childhood adversities may also face barriers to engaging in healthy behaviours (i.e., a lack of access to leisure facilities or a healthy diet), and intervention studies examining the effectiveness of healthy lifestyle choices in childhood adversities should aim to account for societal inequalities.

Factors relating to study design may have also contributed to our findings, including the absence of moderating effects. Whilst the measurement timepoints for lifestyle factors (16 years) and comorbidity (30 years) helped control for reverse causality between outcome and moderators, the developmental period from 16 to 30 years represents a transitional stage of life characterised by greater autonomy (leaving home, leaving education, entering employment, etc.). This may have led to changes in lifestyle behaviours in BCS70 participants, as documented in other cohorts [75–78], although some behaviours such as sleep duration and smoking, may be more stable from adolescence to adulthood [79, 80]. This point is highlighted by the fact that school-time physical activity contributed to a greater extent than extra-curricular sports to our measure of total physical activity, which could evidently change after leaving school.

Sleep duration was also self-reported based on a single night prior to arrival at the assessment centre, which may not be representative of typical sleep patterns [81]. There is also the potential for social desirability bias in self-reported health behaviours, in particular alcohol use (16 years) which was recorded before the legal age of consumption (18 years), and smoking which was measured shortly after the legal age for use in the UK (16 years). These health-risk behaviours have been shown to be under-estimated when self-reported [82–84], highlighting the need to examine both objective and/or repeated measures of lifestyle factors from adolescence to young adulthood. In addition, the cumulative childhood adversity score, as a composite of 33 items, captures substantial variability and explains a large proportion of the variance in the outcomes. In contrast, the single-item moderators capture limited variability and may lack the statistical power to significantly interact with a multidimensional exposure.

As well as examining total levels of physical activity, we also examined different types of physical activity, because one study found consistent participation in team sports, but not individual sports, to reduce depressive symptoms in early adulthood [62]. This indicates that engaging in sports which rely on social cohesion as a core element, may be more beneficial at protecting against mental health problems. This distinction was investigated within our post-hoc analyses, which found that considering team-based sports, individual sports, or skill-based sports separately did not change the main conclusions of the study (i.e., no moderation of the association between childhood adversities and the health outcome categories). Further examination of specific subtypes of physical activity is required to determine the components or types of physical activity that are particularly beneficial for mental health.

### Limitations

Our findings need to be considered within the context of the following limitations. Firstly, despite a comprehensive imputation model, high levels of missing data means that these results should be classified as hypothesis-generating, and the analyses should be replicated in alternative samples to confirm inferences [85]. This is particularly important given the focus of this study, as childhood adversities, poor mental health and risk behaviours have been shown to be associated with higher rates of attrition in longitudinal studies [51, 86–88]. Secondly, each adversity in childhood was dichotomised to denote risk or no risk, which whilst designed to give equal weight to each type of adversity, represents a simplistic view of stressful events. Although an attempt was made to use evidence-based cut-offs, due to the response options given to participants some decisions required subjective deliberation and were based on researcher judgement. To mitigate this, we have been transparent about our dichotomisation criteria (see Supplementary Table 1) to allow other researchers to improve or modify the measure with improved knowledge of what constitutes an adversity. Finally, this study also did not account for any directionality between the co-occurrence of psychological distress and obesity, with some studies suggesting that psychological distress more commonly precedes obesity [89], or vice versa [90].

## Conclusion

We provide further evidence of a dose-dependent relationship between childhood adversities and mental-physical comorbidity in early adulthood. Our findings suggest that lifestyle factors did not operate as significant moderators of the relationship between childhood adversities and mental, physical, and comorbid health outcomes in early adulthood. The strong established association between childhood adversities and psychological distress-obesity comorbidity, should give rise to a more comprehensive investigation into factors which can mitigate the detrimental health consequences of childhood adversities. We further advocate for longitudinal studies to understand how lifestyle factors may dynamically shape the impact of childhood adversity on trajectories of multiple concurrent health conditions across the lifespan, enabling more effective interventions and improved allocation of healthcare resources.

## Electronic supplementary material

Below is the link to the electronic supplementary material.

Supplementary Material 1

## Data Availability

The data that support the findings of this study are available from the UK Data Service, but restrictions apply to the availability of these data, which were used under license for the current study, and so are not publicly available. Data are however available from the authors upon reasonable request and with permission of the UK Data Service.

## Abbreviations

ALSPAC: Avon Longitudinal Study of Parents and Children
BCS70: 1970 British Cohort Study
BMI: Body mass index
exp: Exponentiated
MAR: Missing at random
OR: Odds ratio
UK: United Kingdom
