# Supplementary material for: Examining lifestyle factors as potential moderators of the link between childhood adversity and comorbid psychological distress and obesity in early adulthood
Source: BMC Public Health. 2025 Jul 7;25:2403. doi: 10.1186/s12889-025-23505-6 (PMC12232813; doi:10.1186/s12889-025-23505-6)
Supplement: Supplementary file 1 — Supplementary Material 1 [file 12889_2025_23505_MOESM1_ESM.docx]

**Supplementary Table 1:** Items, dichotomisation strategy and proportional representation of childhood adversities in the BCS70 cohort.

| Item | Age | No risk | N (%) | Risk | N (%) | Report | Miss N (%) |
| --- | --- | --- | --- | --- | --- | --- | --- |
| Natural mother absent | 5 | Relationship to mother figure:   - Natural mother | 12254  (74.7%) | Relationship to mother figure:   - Adopted mother - Stepmother - Foster Mother - Grandmother - Elder sister - Father Cohabitee - Other - No mother | 214 (1.3%) | Parent | 3939 (24.0%) |
|  | 10 |  | 12143 (74.0%) |  | 383 (2.3%) |  | 3881 (23.7%) |
|  | 16 |  | 8206 (50.0%) |  | 338 (2.1%) |  | 7863 (47.9%) |
| Natural father absent | 5 | Relationship to father figure:   - Natural father | 11359 (69.2%) | Relationship to father figure:   - Adopted father - Stepfather - Foster father - Grand father - Elder brother - Mothers’ cohabitee - Other - No father | 1109 (6.8%) | Parent | 3939 (24.0%) |
|  | 10 |  | 10660 (65.0%) |  | 1858 (11.3%) |  | 3889 (23.7%) |
|  | 16 |  | 7057 (43.0%) |  | 1442 (8.8%) |  | 7908 (48.2%) |
| Low parental social class | 5 | Fathers social class (mothers if missing):   - Professional (I) - Managerial and Technical (II) - Skilled occupations (non-manual - III) | 9716 (59.2%) | Both parents:   - Skilled occupations (manual- III) - Partly skilled occupations (IV) - Unskilled occupations (V) - Unemployed | 2239 (13.6%) | Parent | 4452 (27.1%) |
|  | 10 |  | 9863 (60.1%) |  | 2200 (13.4%) |  | 4344 (26.5%) |
|  | 16 |  | 5227 (31.9%) |  | 890 (5.4%) |  | 10290 (62.7%) |
| Low parental education | 5 | Either parent:   - Vocation/typing/apprenticeship - O-level or equivalent - A-level or equivalent - Registered nurse - Degree - Other qualification | 7391 (45.0%) | Both parents:   - No qualifications | 4241 (25.8%) | Parent | 4775 (29.1%) |
|  | 10 |  | 8175 (49.8%) |  | 3665 (22.3%) |  | 4567 (27.8%) |
|  | 16 |  | 5360 (32.7%) |  | 1299 (7.9%) |  | 9748 (59.4%) |
| Damp housing | 10 | No | 10223 (62.3%) | Slight  Moderate  Marked | 2245 (13.7%) | Parent | 3939 (24.0%) |
|  | 16 |  | 7147 (43.6%) |  | 941 (5.7%) | Mother | 8319 (50.7%) |
| Low income | 10 | Household (father + mother) gross income is:   - Above 60% of the median income | 7242 (44.1%) | Household (father + mother) gross income is:   - Below 60% of the median income | 4197 (25.6%) | Parent | 4968 (30.3%) |
|  | 16 |  | 5408 (33.0%) |  | 2353 (14.3%) |  | 8646 (52.7%) |
| Maternal depression risk | 5 | 0-80^th^ percentile on Malaise inventory | 10025 (61.1%) | 81-100^th^ percentile on Malaise inventory | 2199 (13.4%) | Mother | 4183 (25.5%) |
|  | 10 |  | 9180 (56.0%) |  | 2265 (13.8%) |  | 4962 (30.2%) |
|  | 16 |  | 6474 (39.5%) |  | 1370 (8.4%) |  | 8563 (52.2%) |
| Household member illness/ disability | 5 | No household members have developed a serious illness or disability since the study child’s birth | 9989 (60.9%) | Since the study child’s birth, a household member has developed a:   - Severe or prolonged illness - Handicap or disability | 2349 (14.3%) | Parent | 4069 (24.8%) |
|  | 10 |  | 8623 (52.6%) |  | 3719 (22.7%) |  | 4065 (24.8%) |
|  | 16 |  | 5743 (35.0%) |  | 2963 (18.1%) |  | 7701 (46.9%) |
| Social environment risk | 5 | Valid health visitor records during 1^st^ week of life, and any social or environmental risk factors recorded:   - No   Or, valid child health clinic visits during 1^st^ week of life, and any social or environmental risk recorded:   - No | 10707 (65.3%) | Valid health visitor records during 1^st^ week of life, and any social or environmental risk factors recorded:   - Yes   Or, valid child health clinic visit during 1^st^ week of life, and any social or environmental risk recorded:   - Yes | 364 (2.2%) | Health visitor | 5336 (32.5%) |
| Separation from mother | 5 | The child has been separated from the mother:   - From birth until less than 24 hours after birth, or not at all - For less than a day in the first month of life, or not at all - For less than a month in the first 5 years of life, or not at all | 9292 (56.6%) | The child has been separated from the mother:   - From birth until 24 hours after birth - For more than a day in the first month of life - For more than a month in the first 5 years of life | 2963 (18.1%) | Parent | 4152 (25.3%) |
| Lonely at school | 10 | Answered no to both:   - Do you feel lonely at school? (Only available at age 10) - Do you feel sad because you have nobody to play with at school? | 7242 (44.1%) | Answered yes to either:   - Do you feel lonely at school? (Only available at age 10) - Do you feel sad because you have nobody to play with at school? | 4197 (25.6%) | Child | 4968 (30.3%) |
|  | 16 |  | 3727 (22.7%) |  | 304 (1.9%) |  | 12376 (75.4%) |
| Parents not loving | 16 | Answered yes to, my parents:   - Are loving/caring/look after me | 4264 (26.0%) | Answered no to, my parents:   - Are loving/caring/look after me | 1495 (9.1%) | Child | 10648 (64.9%) |
| Non-accidental injury | 5 | Has the child ever had any injury considered or suspected to be “non-accidental?   - No | 10215 (62.3%) | Has the child ever had any injury considered or suspected to be “non-accidental?   - Yes - No, but at risk | 356 (2.2%) | Health visitor | 5836 (35.6%) |
| Peer victimisation | 10 | Other children say nasty things about you:   - No - Don’t know | 8051 (49.1%) | Other children say nasty things about you:   - Yes | 3336 (20.3%) | Child | 5020 (30.6%) |
|  | 16 |  | 3292 (20.1%) |  | 739 (4.5%) |  | 12376 (75.4%) |
| Hit by parent | 16 | Told off by parent in last month?   - No - Yes, but not hit | 5406 (32.9%) | Told off by parent in last month?   - Yes, hit by parent | 169 (1.0%) | Child | 10832 (66.0%) |
| Treated unfairly | 16 | Treated unfairly in the last 12 months, due to:   - Never been treated unfairly   And, picked on by teacher, more than others:   - No - Nobody in class gets picked on - Other | 5656 (34.5%) | Treated unfairly in the last 12 months, due to:   - Sex - Skin colour - Dress - Family - Speech - Religion - Other reason - Not known why   Or, picked on by teacher, more than others:   - Yes | 2139 (13.0%) | Child | 8612 (52.5%) |
| Sexual abuse | 16 | Have you ever had any unwelcomed sexual approaches made to you?   - No | 4897 (29.8%) | In the past 12 months, someone used force for any reason.   - Yes, to make me have sex when I didn’t want to - Yes, to make me do petting   Have you ever had any unwelcomed sexual approaches made to you?   - Yes | 802 (4.9%) | Child | 10708 (65.3%) |

| Team-based sports | Baseball, basketball, cricket, football, hockey, netball, rounders, rugby, volleyball |
| --- | --- |
| Individual-based sports | Aerobics, track/field, badminton, canoe, cross-country, cycling, dancing, gymnastics, horse-riding, jogging, fitness exercise, roller/ice-skating, rowing, sailing, scrambling, skiing, squash, swimming, table tennis, tennis, walking, water-skiing, weight training, wind surfing |
| Skill-based sports | Billiards, darts, fishing, pool, shooting, snooker |

**Supplementary Table 2:** Types of sport included within each physical activity category.

**Supplementary Table 3:** Food items within Mediterranean diet food groups, and categorisation criteria

|  |  | Amount consumed (g/day) | | |
| --- | --- | --- | --- | --- |
| Food group | Items | 0 | 1 | 2 |
| Fruit | Unsweetened fruit juice  Fresh fruit (not citrus/apple pear)  Citrus fruits  Apples and pears  Tomatoes | < 150 | 150-300 | > 300 |
| Vegetables | Salad vegetables  Fresh and frozen vegetables  Canned or processed vegetables  Green vegetables  Carrots | < 100 | 100-250 | > 250 |
| Legumes | Peas  Baked beans | < 70 | 70-140 | > 140 |
| Cereals | White bread  Wholemeal bread  Brown wheatgerm granary bread  Other breads  High fibre breakfast cereals  Other breakfast cereals (unsweetened)  Pasta, rice, cereals | < 130 | 130-195 | > 195 |
| Fish | Fish  Fish dishes | < 100 | 100-250 | > 250 |
| Meat | Bacon and ham  Poultry and poultry dishes  Offal and dishes  Canned meat  Meat pies  Beef and veal  Lamb  Pork  Coated chicken products  Burgers and kebabs  Sausages | > 120 | 80-120 | < 80 |
| Dairy | Cheese  Eggs  Eggs and cheese dishes  Canned meat  Butter  Whole milk  Semi-skimmed milk  Other milk and cream  Yoghurt  Cottage cheese  Skimmed milk  Milk puddings | > 270 | 180-270 | < 180 |

| Items | Questions |
| --- | --- |
| 1 | Do you often have backache? |
| 2 | Do you feel tired most of the time? |
| 3 | Do you often feel depressed? |
| 4 | Do you often have bad headaches? |
| 5 | Do you often get worried about things? |
| 6 | Do you usually have great difficulty in falling or staying asleep? |
| 7 | Do you usually wake unnecessarily early in the morning? |
| 8 | Do you wear yourself out worrying about your health? |
| 9 | Do you often get into a violent rage? |
| 10 | Do people annoy and irritate you? |
| 11 | Have you at times had a twitching of the face, head or shoulders? |
| 12 | Do you suddenly become scared for no good reason? |
| 13 | Are you scared to be alone when there are not friends near you? |
| 14 | Are you easily upset or irritated? |
| 15 | Are you frightened of going out alone or of meeting people? |
| 16 | Are you constantly keyed up and jittery? |
| 17 | Do you suffer from indigestion? |
| 18 | Do you suffer from an upset stomach? |
| 19 | Is your appetite poor? |
| 20 | Does every little thing get on your nerves and wear you out? |
| 21 | Does your heart often race like mad? |
| 22 | Do you often have bad pain in eyes? |
| 23 | Are you troubled with rheumatism or fibrosis? |
| 24 | Have you ever had a nervous breakdown? |

Note: The response options were "yes" and "no" at age 30 in BCS70.

**Supplementary Table 4:** Malaise Inventory items.

**Supplementary Figure 1**: The total number of missing childhood items per number of participants in the study sample.


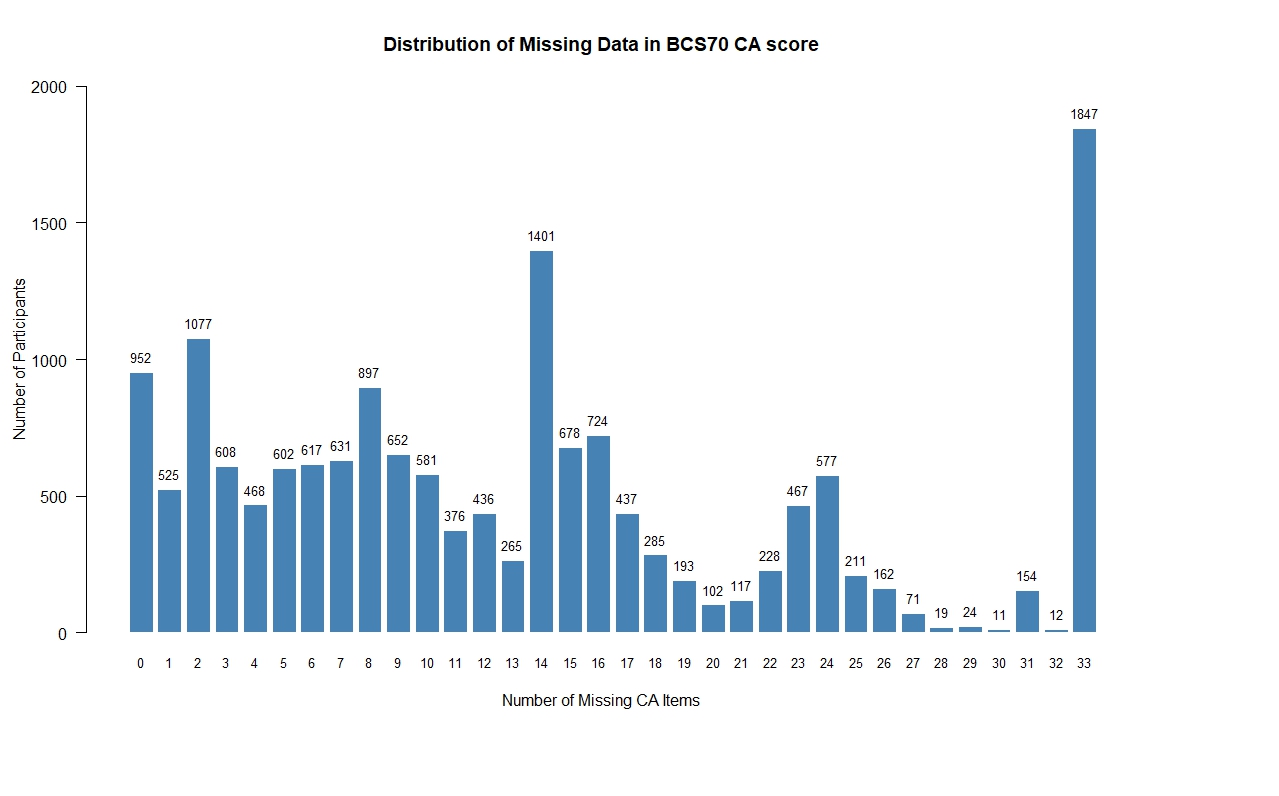


**Supplementary Figure 2**: Imputation model diagram


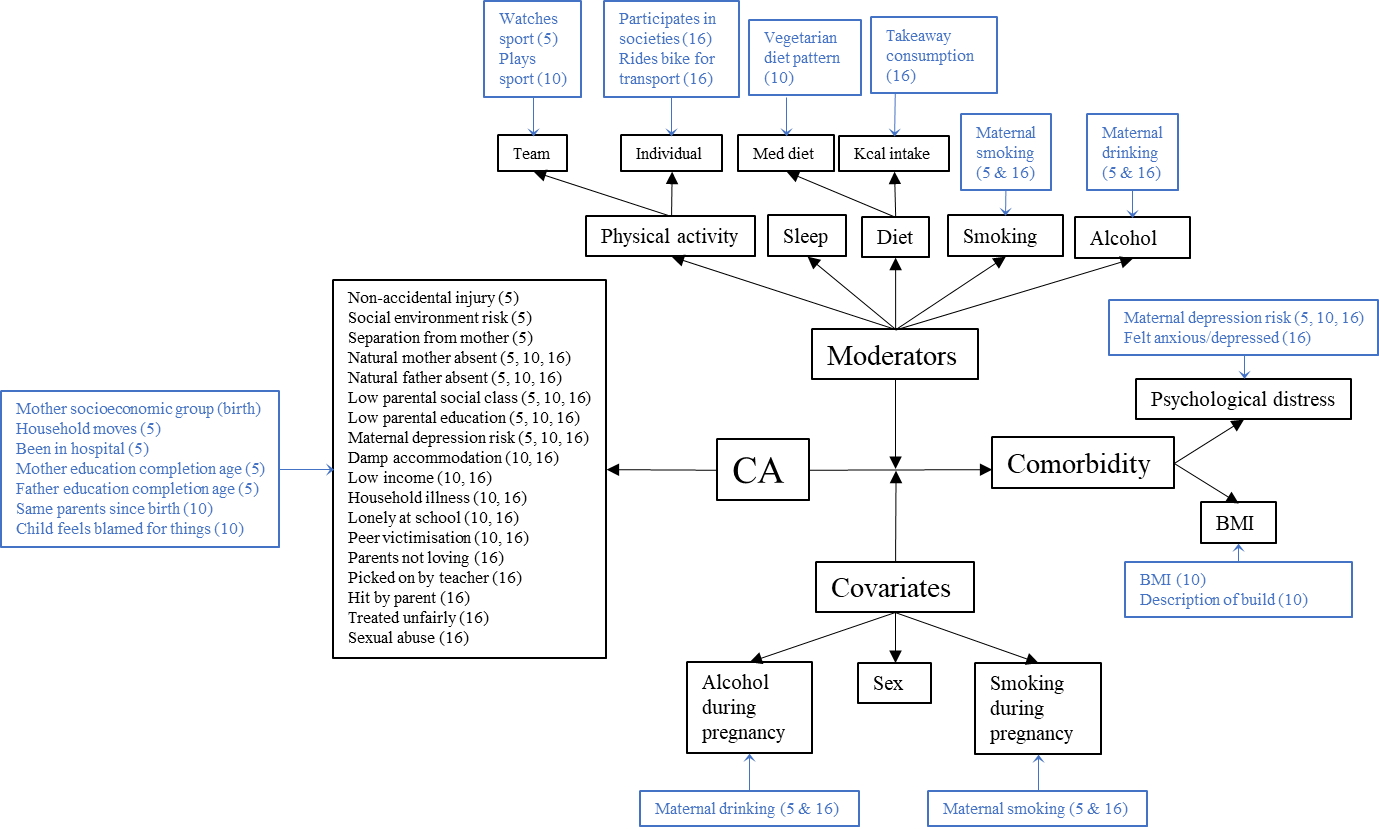


Note: Items in black are items included in the imputation analyses models, whilst items in blue (auxiliary variables) were only included in the imputation model. Auxiliary variables were required to have a correlation of above 0.1 with the respected analysis items. Ages (years) at which auxiliary variables were recorded and included are stated in the diagram.

**Supplementary Table 5:** Details of missing rates and the specification of imputation methods per parameter

| **Parameter** | **Miss %** | **Method** | **Predictors** |
| --- | --- | --- | --- |
| Childhood adversities | 94.2% | 1) PMM (item-level) 2) Passive imputation | CA items, moderators, covariates, outcome, auxiliary |
| Physical activity | 71.0% | 1) PMM (item-level) 2) Passive imputation | Exposure, covariates, outcomes, auxiliary |
| Mediterranean diet | 73.9% | 1) PMM (item-level) 2) Passive imputation | Exposure, covariates, outcomes, auxiliary |
| Energy consumption | 74.4% | PMM | Exposure, covariates, outcomes, auxiliary |
| Sleep duration | 71.8% | 1) PMM (item-level) 2) Passive imputation | Exposure, covariates, outcomes, auxiliary |
| Alcohol consumption | 66.3% | PMM | Exposure, covariates, outcomes, auxiliary |
| Cigarette smoking | 66.5% | PMM | Exposure, covariates, outcomes, auxiliary |
| Sex | 0.0% | PMM | Exposure, moderators, outcomes, interactions |
| Maternal drinking | 28.6% | PMM | Exposure, moderators, outcomes, auxiliary, interactions |
| Maternal smoking | 0.5% | PMM | Exposure, moderators, outcomes, auxiliary, interactions |
| Psychological distress | 38.5% | 1) PMM (item-level) 2) Passive imputation | Exposure, moderators, covariates, auxiliary, interactions |
| BMI | 42.7% | 1) PMM (item-level) 2) Passive imputation | Exposure, moderators, covariates, auxiliary, interactions |
| Comorbidity categories | 43.2% | Passive imputation | N/A |

Note: Exposure = childhood adversities, moderators = physical activity, Mediterranean diet, energy consumption, alcohol consumption, cigarette smoking, outcome = comorbidity categories, covariates = sex, maternal drinking, maternal smoking, interactions = exposure*lifestyle factors, CA items = childhood adversity items, auxiliary = auxiliary variables (see Supplementary Figure 2), PMM = predictive mean matching.

|  | Controls  (N=1275) | Psychological Distress Only  (N=118) | Obesity Only  (N=130) | Comorbidity Risk  (N=22) |
| --- | --- | --- | --- | --- |
| **Childhood Adversities** |  |  |  |  |
| Number of stressors | 4.30 (3.12) | 5.89 (3.38) | 5.46 (2.66) | 7.00 (2.58) |
| Missing (%) | 838 (65.7%) | 82 (69.5%) | 84 (64.6%) | 18 (81.8%) |
| Prorated scale | 4.70 (3.24) | 6.41 (3.87) | 5.88 (3.22) | 8.74 (4.24) |
| **Physical Activity** |  |  |  |  |
| Sessions per week | 6.37 (4.84) | 6.61 (5.47) | 5.98 (4.56) | 4.97 (4.15) |
| **Mediterranean diet** |  |  |  |  |
| Score | 3.94 (1.86) | 3.77 (1.86) | 3.84 (1.69) | 3.36 (1.50) |
| **Energy Consumption** |  |  |  |  |
| Daily calorie intake | 2540 (829) | 2550 (950) | 2400 (874) | 2630 (932) |
| **Sleep duration** |  |  |  |  |
| Hours | 8.75 (1.13) | 8.70 (1.47) | 8.60 (1.27) | 8.25 (1.37) |
| **Cigarette smoking (weekly)** |  |  |  |  |
| Non-smoker | 1017 (79.8%) | 81 (68.6%) | 99 (76.2%) | 19 (86.4%) |
| 0-10 | 97 (7.6%) | 16 (13.6%) | 13 (10.0%) | 1 (4.5%) |
| 11-40 | 101 (7.9%) | 12 (10.2%) | 12 (9.2%) | 0 (0%) |
| 41+ | 60 (4.7%) | 9 (7.6%) | 6 (4.6%) | 2 (9.1%) |
| **Alcohol consumption (frequency)** |  |  |  |  |
| Non-drinker | 105 (8.2%) | 12 (10.2%) | 8 (6.2%) | 2 (9.1%) |
| Special occasions only | 367 (28.8%) | 30 (25.4%) | 41 (31.5%) | 5 (22.7%) |
| Once a month | 197 (15.5%) | 17 (14.4%) | 24 (18.5%) | 3 (13.6%) |
| Once a week | 384 (30.1%) | 31 (26.3%) | 33 (25.4%) | 4 (18.2%) |
| 2 or more times a week | 222 (17.4%) | 28 (23.7%) | 24 (18.5%) | 8 (36.4%) |
| **Sex** |  |  |  |  |
| Male | 499 (39.1%) | 35 (29.7%) | 51 (39.2%) | 7 (31.8%) |
| Female | 776 (60.9%) | 83 (70.3%) | 79 (60.8%) | 15 (68.2%) |
| **Malaise Inventory** |  |  |  |  |
| Sum score | 2.30 (2.10) | 10.6 (2.67) | 2.59 (2.02) | 10.0 (2.08) |
| **Body Mass Index** |  |  |  |  |
| Kg/M^2^ | 23.6 (2.89) | 23.0 (3.27) | 33.4 (3.30) | 36.3 (4.58) |

**Supplementary Table 6:** Sample characteristics with complete outcome, moderator, and covariate data

Note: Continuous (Mean; SD) and categorical (Counts; %) data types from participants with complete outcome, moderator and covariate data, stratified by outcome group. Missing % in childhood adversities displays the number of participants with at least 1 missing childhood adversity item. The prorated scale was calculated in participants with valid responses for at least 80% of all childhood adversity items.

**Supplementary Table 7:** Results of the multinomial regression model without interactions.

|  |  | No interaction model | | | | |
| --- | --- | --- | --- | --- | --- | --- |
| Outcome | Predictor | Odds Ratio (OR) | Std.Error | P-value | 95% CI (Lower) | 95% CI (Upper) |
| Psychological  Distress only | Childhood adversities | 1.110 | 0.009 | <0.001 | 1.090 | 1.130 |
|  | Sex (Female) | 1.334 | 0.057 | <0.001 | 1.193 | 1.492 |
|  | Maternal drinking | 0.944 | 0.059 | 0.333 | 0.839 | 1.062 |
|  | Maternal smoking | 1.049 | 0.015 | 0.001 | 1.019 | 1.081 |
| Obesity only | Childhood adversities | 1.045 | 0.009 | <0.001 | 1.028 | 1.063 |
|  | Sex (Female) | 0.872 | 0.069 | 0.047 | 0.762 | 0.998 |
|  | Maternal drinking | 1.039 | 0.058 | 0.514 | 0.926 | 1.165 |
|  | Maternal smoking | 1.067 | 0.017 | <0.001 | 1.033 | 1.102 |
| Comorbidity risk | Childhood adversities | 1.158 | 0.018 | <0.001 | 1.118 | 1.199 |
|  | Sex (Female) | 1.426 | 0.152 | 0.020 | 1.058 | 1.922 |
|  | Maternal drinking | 1.016 | 0.123 | 0.896 | 0.797 | 1.295 |
|  | Maternal smoking | 1.148 | 0.037 | <0.001 | 1.067 | 1.234 |

**Supplementary Table 8:** Results of the multinomial regression models without interactions in individuals with complete outcome and covariate data.

|  |  | No interaction model – Sensitivity analysis | | | | |
| --- | --- | --- | --- | --- | --- | --- |
| Outcome | Predictor | Odds Ratio (OR) | Std.Error | P-value | 95% CI (Lower) | 95% CI (Upper) |
| Psychological  Distress only | Childhood adversities | 1.139 | 0.027 | <0.001 | 1.080 | 1.201 |
|  | Sex (Female) | 1.509 | 0.212 | 0.053 | 0.995 | 2.287 |
|  | Maternal drinking | 1.161 | 0.050 | 0.003 | 1.052 | 1.281 |
|  | Maternal smoking | 0.919 | 0.159 | 0.593 | 0.673 | 1.254 |
| Obesity only | Childhood adversities | 1.106 | 0.026 | <0.001 | 1.050 | 1.165 |
|  | Sex (Female) | 0.982 | 0.190 | 0.923 | 0.677 | 1.424 |
|  | Maternal drinking | 1.061 | 0.049 | 0.227 | 0.964 | 1.168 |
|  | Maternal smoking | 0.816 | 0.147 | 0.167 | 0.611 | 1.089 |
| Comorbidity risk | Childhood adversities | 1.332 | 0.055 | <0.001 | 1.197 | 1.482 |
|  | Sex (Female) | 1.371 | 0.468 | 0.500 | 0.548 | 3.430 |
|  | Maternal drinking | 0.950 | 0.119 | 0.665 | 0.753 | 1.199 |
|  | Maternal smoking | 1.062 | 0.385 | 0.875 | 0.500 | 2.258 |

**Supplementary Table 9:** Interaction terms from multinomial logistic regression models.

|  |  |  | | Interaction models | | | | |
| --- | --- | --- | --- | --- | --- | --- | --- | --- |
| Outcome | Predictor | Odds Ratio (OR) | Std.Error | P-value | Q-value | | 95% CI (Lower) | 95% CI (Upper) |
| Psychological  distress | CA:PA | 0.997 | 0.002 | 0.249 | 0.420 | | 0.993 | 1.002 |
|  | CA:MD | 1.002 | 0.005 | 0.712 | 0.857 | | 0.992 | 1.012 |
|  | CA:CI | 0.999 | 0.008 | 0.945 | 0.981 | | 0.984 | 1.015 |
|  | CA:SD | 1.001 | 0.007 | 0.859 | 0.949 | | 0.987 | 1.016 |
|  | CA:AC | 1.002 | 0.006 | 0.802 | 0.932 | | 0.989 | 1.014 |
|  | CA:SB | 1.000 | 0.008 | 0.970 | 0.986 | | 0.984 | 1.016 |
| Obesity only | CA:PA | 0.998 | 0.002 | 0.408 | 0.616 | | 0.994 | 1.003 |
|  | CA:MD | 1.002 | 0.005 | 0.641 | 0.808 | | 0.992 | 1.013 |
|  | CA:CI | 1.002 | 0.010 | 0.844 | 0.944 | | 0.983 | 1.021 |
|  | CA:SD | 1.002 | 0.006 | 0.781 | 0.923 | | 0.989 | 1.015 |
|  | CA:AC | 1.003 | 0.007 | 0.669 | 0.831 | | 0.989 | 1.017 |
|  | CA:SB | 0.995 | 0.008 | 0.523 | 0.706 | | 0.978 | 1.011 |
| Comorbidity  risk | CA:PA | 0.994 | 0.006 | 0.275 | 0.460 | | 0.982 | 1.005 |
|  | CA:MD | 1.004 | 0.011 | 0.705 | 0.854 | | 0.983 | 1.026 |
|  | CA:CI | 1.012 | 0.018 | 0.511 | 0.706 | | 0.976 | 1.050 |
|  | CA:SD | 1.000 | 0.013 | 0.995 | 0.995 | | 0.974 | 1.027 |
|  | CA:AC | 1.012 | 0.014 | 0.411 | 0.616 | | 0.984 | 1.040 |
|  | CA:SB | 0.997 | 0.019 | 0.883 |  | 0.949 | 0.960 | 1.036 |

Note: PA=physical activity, MD=Mediterranean diet, CI=calorie intake (z-score), SD=sleep duration, AC=alcohol consumption, SB=smoking behaviour.

|  |  | Interaction models – Sensitivity analyses | | | | |
| --- | --- | --- | --- | --- | --- | --- |
| Outcome | Predictor | Odds Ratio (OR) | Std.Error | P-value | 95% CI (Lower) | 95% CI (Upper) |
| Psychological  distress | CA:PA | 1.002 | 0.005 | 0.772 | 0.991 | 1.012 |
|  | CA:MD | 0.994 | 0.015 | 0.704 | 0.965 | 1.024 |
|  | CA:EC | 1.007 | 0.062 | 0.909 | 0.891 | 1.138 |
|  | CA:SD | 1.027 | 0.017 | 0.118 | 0.993 | 1.062 |
|  | CA:AC | 1.013 | 0.020 | 0.518 | 0.974 | 1.054 |
|  | CA:SB | 1.015 | 0.029 | 0.602 | 0.959 | 1.075 |
| Obesity only | CA:PA | 1.003 | 0.005 | 0.514 | 0.993 | 1.014 |
|  | CA:MD | 1.015 | 0.015 | 0.327 | 0.986 | 1.044 |
|  | CA:EC | 1.055 | 0.056 | 0.332 | 0.947 | 1.177 |
|  | CA:SD | 1.013 | 0.018 | 0.451 | 0.979 | 1.049 |
|  | CA:AC | 1.013 | 0.020 | 0.520 | 0.974 | 1.053 |
|  | CA:SB | 1.002 | 0.031 | 0.958 | 0.943 | 1.064 |
| Comorbidity  risk | CA:PA | 0.988 | 0.013 | 0.368 | 0.963 | 1.014 |
|  | CA:MD | 0.976 | 0.031 | 0.442 | 0.918 | 1.038 |
|  | CA:EC | 0.952 | 0.155 | 0.751 | 0.703 | 1.289 |
|  | CA:SD | 1.011 | 0.035 | 0.760 | 0.944 | 1.083 |
|  | CA:AC | 0.984 | 0.041 | 0.701 | 0.908 | 1.067 |
|  | CS:SB | 0.938 | 0.077 | 0.408 | 0.806 | 1.092 |

**Supplementary Table 10:** Interaction terms from multinomial regression models applied to individuals with complete outcome, moderator, and covariate data.

Note: PA=physical activity, MD=Mediterranean diet, CI=calorie intake (z-score), SD=sleep duration, AC=alcohol consumption, SB=smoking behaviour.
